# Novel Hertz Contact Intravascular Lithotripsy: Could We Achieve More in Balloon-Based Calcium Modification?

**DOI:** 10.3390/jcm15051802

**Published:** 2026-02-27

**Authors:** Andreas Mitsis, Elina Khattab, Matthaios Didagelos, Konstantinos C. Theodoropoulos, Aggeliki D. Mavrogianni, Antonios Ziakas, Nikolaos Fragakis, George Kassimis

**Affiliations:** 1 Cardiology Department, Nicosia General Hospital, State Health Services Organization, Nicosia 2029, Cyprus; andymits7@gmail.com (A.M.); khattab_elina@outlook.com (E.K.); 2 First Cardiology Department, AHEPA University Hospital, Aristotle University of Thessaloniki, 54636 Thessaloniki, Greece; manthosdid@yahoo.gr (M.D.); ktheod2005@hotmail.com (K.C.T.); tonyziakas@hotmail.com (A.Z.); 3 Cardiology Department, General Hospital G. Papanikolaou, 57010 Thessaloniki, Greece; amavrogianni@yahoo.com; 4 Second Cardiology Department, General Hospital ‘Hippokration’, Aristotle University of Thessaloniki, 54642 Thessaloniki, Greece; fragakis.nikos@gmail.com

**Keywords:** coronary calcification, balloon-based calcium modification, Hertz-contact lithotripsy, intravascular lithotripsy, non-compliant balloon, ultra-high-pressure balloon, cutting balloon, scoring balloon, complex percutaneous coronary intervention

## Abstract

Severe coronary artery calcification (CAC) remains a major challenge in percutaneous coronary intervention (PCI), driving stent under-expansion and higher rates of restenosis and adverse events. Balloon-based calcium modification remains central to lesion preparation, with the available tools ranging from high-pressure non-compliant balloons and ultra-high-pressure balloons to cutting, scoring, and intravascular lithotripsy (IVL) balloons. While traditional IVL has advanced the field by permitting circumferential fracture of deep calcium through acoustic shockwaves, important drawbacks persist, including problems in deliverability, energy distribution, and questionable efficacy in nodular or eccentric calcium. This review examines all contemporary balloon-based modification strategies and introduces the novel Hertz-contact IVL (HC-IVL), a new technology designed to transmit mechanical energy through direct contact rather than shockwave propagation. Based on Hertzian mechanics, this device may facilitate more focused energy delivery, improved lesion crossing, and enhanced calcium fracture in complex morphologies. A detailed comparison between HC-IVL and standard IVL is provided, along with a proposed algorithm for device selection. Taking into consideration the limitations of current tools, HC-IVL represents a promising mechanistic innovation in balloon-based calcium modification, warranting further validation in randomized, imaging-guided clinical studies.

## 1. Introduction

Coronary artery calcification (CAC) represents one of the most significant challenges in contemporary percutaneous coronary intervention (PCI). As populations age and the prevalence of diabetes mellitus and chronic kidney disease rises, heavily calcified lesions appear with increasing frequency in daily practice. The presence of extensive calcium is a strong predictor of stent under-expansion [1], which in turn is closely linked to in-stent restenosis, stent thrombosis, and target lesion failure [2]. Effective modification of coronary calcium is therefore essential to achieving optimal stent deployment and improving clinical outcomes [3].

Besides rotational and orbital atherectomy, balloon-based lesion preparation remains the most widely used method and is often a first-line approach for coronary calcium modification. Non-compliant (NC) and ultra-high-pressure (UHP) balloons [4], scoring and cutting balloons [5], and more recently intravascular lithotripsy (IVL) have expanded the interventional toolkit, enabling operators to manage a broader spectrum of complex calcified lesions more effectively [6,7]. Specifically, IVL, based on acoustic shockwave emission within a balloon catheter, has become a game-changer modality capable of modifying deep and circumferential calcium with an overall favorable safety profile [8]. However, limitations persist, including limited deliverability in severely stenotic or tortuous anatomy, particularly for bulkier balloon-based devices, reliance on shockwave propagation through fluid rather than direct calcium contact, and variable effectiveness in eccentric or nodular calcification [9].

These gaps have spurred interest in next-generation approaches to balloon-based plaque modification. Hertz Contact Intravascular Lithotripsy (HC-IVL) is an emerging technology designed to deliver mechanical energy directly through contact interfaces predicted by Hertzian mechanics. This method aims to achieve more localized and efficient calcium fracture, potentially reducing the dependance on extreme pressures while expanding the spectrum of treatable lesion morphologies. In this review, we examine the full range of available balloon-based modification tools, compare the underlying mechanisms of conventional IVL and HC-IVL, and explore whether this novel technology may enable a new level of performance in calcium modification.

## 2. Understanding Coronary Calcification

Coronary artery calcification is not a uniform entity; rather, it encompasses a spectrum of morphologies with distinct mechanical behaviors and procedural implications [10]. Intravascular imaging with the use of optical coherence tomography (OCT) and intravascular ultrasound (IVUS) has refined the understanding of these patterns and enabled accurate prediction of device performance and stent expansion (Table 1).

### 2.1. Superficial vs. Deep Calcium

Superficial calcium is defined as when the leading edge of acoustic shadow lies within the shallow 50% of the plaque and media thickness, directly opposes balloon expansion and is the primary determinant of balloon compliance. Superficial calcium is more amenable to surface-modifying tools such as scoring or cutting balloons and to superficial fracturing with atherectomy [11]. Deep calcium, is typically thicker, distributed circumferentially and lies within deepest 50% of the plaque and media thickness. It resists luminal expansion despite high-pressure balloon inflations and may require technologies capable of transmitting energy across the vessel wall, such as IVL [12].

### 2.2. Calcified Nodules vs. Circumferential Calcium

Calcified nodules and circumferential calcium represent distinct phenotypes with markedly different mechanical behaviors. Calcified nodules are focal, protruding lesions that disrupt the luminal contour and create a localized resistance to expansion, often leading to balloon slippage or fracture, irregular stent geometry, and incomplete apposition [13]. In contrast, circumferential calcium forms a continuous rigid ring that uniformly constrains vessel expansion and significantly increases the force required for luminal gain. While nodular calcium is associated with eccentric stress concentration and unpredictable fracture patterns [14], circumferential calcium primarily causes global under-expansion and elastic recoil. Both morphologies are strongly linked to procedural failure, but thick and extensive circumferential calcium poses a greater challenge to achieving adequate stent expansion with conventional balloon-based strategies [15].

### 2.3. Calcific Length and Thickness

Long segments (>5 mm) and thick calcium (>0.5 mm) are independently associated with poor balloon compliance and reduced fracture probability with conventional tools [16]. Successful modification in these lesions often requires the combination of calcium-ablation techniques (rotational atherectomy, orbital atherectomy) [17] with sequencing of different balloon-based technologies or debulking devices (e.g., excimer laser) [18]. Beyond mechanical rigidity, coronary calcification often coexists with plaque vulnerability and perivascular inflammation, as demonstrated by recent CCTA-based studies linking calcific burden with adverse plaque features and inflammatory signaling [19].

### 2.4. How Balloons Interact with Calcium

The interaction between a balloon and a calcified coronary lesion is determined by the principles of force transmission and the intrinsic structural characteristics of calcified tissue [20]. Vessel expansion during balloon inflation occurs via both direct mechanical loading and indirect forces from the deformation of the lesion. Direct radial compression results from the balloon pressing against the luminal surface of the plaque [21]. This component reflects the immediate transfer of inflation pressure to the lesion, which is essential for cracking superficial or thin calcium. Indirect radial forces arise from changes in balloon geometry during inflation. As the balloon expands, its shape creates additional stress concentrations at focal points of contact [22]. Both of these forces are significantly influenced by the balloon’s design, compliance, and the plaque’s morphology [23]. Together, these mechanisms define how efficiently a balloon can deform or fracture calcium. In heavily calcified lesions, direct pressure alone is often insufficient, as calcium is inelastic and capable of redistributing force away from the target segment. Indirect forces, therefore, become crucial, especially for specialty balloons that are engineered to maximize focal stress [24].

## 3. Contemporary Balloon-Based Calcium Modification Tools

### 3.1. Non-Compliant Balloons

NC balloons represent the most basic and widely used tool for coronary lesion preparation in the setting of calcified plaque during PCI [25]. These balloons are manufactured from thick, rigid polymeric materials that exhibit minimal diameter increase with escalating inflation pressure, allowing the delivery of high pressures to overcome plaque resistance and facilitate stent expansion [15,26]. Standard NC balloons typically tolerate ≤ 20–24 atm [27]. In calcified lesions, NC balloons act primarily by applying uniform radial force, leading to elastic deformation of the vessel and superficial calcium compression rather than true calcium fracture [28].

In severely calcified lesions, traditional NC balloons are often insufficient as standalone tools, frequently requiring adjunctive strategies to achieve optimal stent expansion [5]. Intravascular imaging studies using OCT and IVUS demonstrated that high-pressure NC balloon inflation rarely produces fractures in thick (>0.5 mm) or deep calcium sheets, which are the main determinants of stent under-expansion. As a result, lesion resistance often persists even at very high inflation pressures, leading to inadequate lumen gain and predisposing to suboptimal stent deployment [29].

However, the escalation of inflation pressure in more than 20–25 atmospheres substantially raise safety issues and increase the risk of vessel injury, including deep dissections, intramural hematoma, and coronary perforation, particularly in eccentric or long calcified lesions [30,31]. Registry data and pooled analyses have shown that the use of very high balloon pressures is independently associated with a higher incidence of perforation, especially in elderly patients and in vessels with reduced compliance due to circumferential calcification [32,33].

Additionally, NC balloons demonstrate significant deliverability limitations in severely calcified or tortuous anatomy. Tight calcific stenoses may restrict balloon crossing, necessitating downsizing or alternative modification strategies, which can prolong procedures and increase contrast and radiation exposure [34]. These limitations are particularly evident in lesions with nodular calcifications, where focal protrusions into the lumen create uneven radial force and predispose to balloon slippage or dog-boning rather than effective plaque modification [35]. Therefore, despite the fact that NC balloons remain a fundamental first-line tool for lesion preparation, their reliance on extreme pressure rather than controlled calcium modification limits their effectiveness in severe coronary calcification and exposes patients to an increased risk of mechanical complications, underscoring the need for adjunctive or alternative balloon-based calcium modification technologies [36].

### 3.2. Ultra-High-Pressure Balloons (UHP)

UHP balloons are characterized by a specialized double-layer, twin-wall design that permits consistent expansion at substantially higher inflation pressures compared with standard NC balloons [37]. This bi-layer design reduces the “dog-boning” effect of uneven balloon expansion and allows direct delivery of intense radial force to calcified plaque, helping the lumen expansion in resistant lesions [21]. The UHP balloons like the OPN™ NC can tolerate pressures up to 35 atm or higher for particularly resistant calcified lesions [38]. Clinical studies have demonstrated that such UHP balloons can achieve adequate lesion dilatation and stent expansion comparable to alternative calcium modification technologies, including IVL, while maintaining acceptable procedural success rates [37,39]. In the prospective, multicenter, randomized non-inferiority VICTORY Trial (Intravascular Lithotripsy vs. Super-High-Pressure Non-Compliant Balloon for Treatment of Calcified and Refractory Coronary Lesions-NCT05346068) presented at TCT 2025, OPN NCB achieved stent expansion and safety outcomes similar to IVL, emerging as a more accessible and cost-effective option for OCT-guided treatment of severely calcified coronary lesions. Procedural success and safety outcomes were comparable between strategies, with no excess of major complications. These findings suggest that super-high-pressure balloons represent an effective alternative to IVL in selected heavily calcified lesions [40].

Prospective randomized results from the ISAR-CALC trial indicate that in patients with severely calcified coronary artery lesions, preparation with a UHP versus a scoring balloon was associated with comparable stent expansion on intravascular imaging and a trend towards improved angiographic performance [41]. Despite higher nominal pressures, reported perforation rates with UHP balloons remain low in appropriately selected lesions [4].

### 3.3. Cutting Balloons

Cutting balloons are specialized devices featuring microsurgical blades arranged longitudinally on the balloon surface, which create controlled incisions in atherosclerotic plaque during inflation to promote vessel lumen expansion before stent deployment [3]. When inflated in a calcified lesion, the micro-blades of a cutting balloon produce shallow longitudinal cuts into the plaque surface decreasing elastic recoil and enhancing vessel compliance [3]. This targeted force approach contrasts with pure radial expansion, as the blades focus stress at specific plaque sites instead of depending only on balloon pressure to crack stiff elements, potentially yielding superior modification for select lesion types [42]. Clinical evidence indicates that cutting balloons yield larger immediate lumen gains and better minimal stent areas than standard NC balloons for focal, moderately calcified lesions, as shown in the COPS randomized trial [42]. In the recently presented at TCT 2025 ShortCUT trial (NCT06089135), cutting balloon angioplasty is a reasonable, safe, and less costly alternative to IVL in IVUS-guided PCI. Procedural success rates were high in both groups, with no significant safety differences. However, IVL demonstrated more consistent results in lesions with deeper or more circumferential calcium [43]. Moreover, a recent finite element analysis showed that in 180° calcified coronary lesions, orienting two cutting-balloon blades toward the calcification significantly increased stress within the calcium while reducing stress on the adjacent arterial wall, favoring effective fracture with less vessel injury. Importantly, the use of slightly undersized balloons (0.25–0.5 mm below reference diameter) further enhanced calcification expansion while potentially lowering the risk of dissection and perforation, even at higher inflation pressures [44,45]. However, there are potential risks including blade fracture, stent damage, or entrapment and the judicious use of cutting balloons is an effective strategy when NC balloons fail [46].

Intravascular imaging in current practice shows that cutting balloons’ controlled incisions improve stent expansion and apposition, especially with IVUS guidance, though large-scale randomized evidence is still limited [47]. However, cutting balloons have several limitations due to their larger device profile and reduced deliverability, complicating delivery through tortuous or severely narrowed calcified segments, which has traditionally restricted their role in complex calcified PCI procedures [3]. Furthermore, cutting balloons carry risks including vessel dissection and perforation, particularly when oversized or used for aggressive dilation without imaging guidance [42]. Ongoing trials like CUPID seek to deliver further data on the relative efficacy and safety of advanced cutting balloons (e.g., Wolverine™) compared to NC balloons, potentially clarifying their optimal use in preparing calcified lesions [48].

### 3.4. Scoring Balloons

Scoring balloons are specialized balloon catheters with external scoring elements, like nitinol or silicone wires, that focus dilation forces at specific points to allow more precise plaque modification than conventional NC balloons [49]. These scoring elements secure the balloon against the vessel wall and generate targeted stress points within the lesion, lowering the inflation pressure needed for plaque fracture while minimizing vessel injury [49]. Biomechanical studies show that scoring balloons generate elevated principal stress at targeted plaque sites, promoting micro-fractures, particularly in fibro-calcific areas, while limiting radial force on unaffected vessel segments [50]. Clinical registry data from the Naviscore study with the novel Naviscore scoring balloon effectively showed reduction in the initial stenosis verified by OCT, achieving over 90% procedural success rates in moderate-to-severe calcified lesions [51]. Comparative results from the ISAR-CALC randomized trial indicate that scoring balloons provide similar stent expansion metrics to UHP balloons in severely calcified coronary lesions, though UHP balloons showed trends toward larger minimum lumen diameters and reduced residual stenosis [41]. Prospective and real-world studies show that scoring balloon angioplasty yields superior angiographic lumen gains and higher rates of optimal results compared to non-scoring balloon pre-dilation, with reduced severe dissections and equivalent flow outcomes [52]. Meta-analyses have shown that specialized balloons (including scoring balloons) used for lesion preparation in calcified PCI are associated with a trend toward lower risk of major adverse cardiac events (MACE) compared with controls [5]. Scoring balloons typically exhibit a low rate of major complications like coronary perforation when properly applied, owing to their controlled expansion that curbs barotrauma compared to high-pressure dilation by itself [52]. Randomized device trials (e.g., Wedge™ NC scoring balloon vs. ScoreFlex™) demonstrate non-inferior procedural success rates and equally low complication rates, affirming the utility of scoring balloons for diverse coronary lesions [53]. However, deliverability remains more limited compared with standard NC balloons in complex anatomy, often requiring supplementary techniques (e.g., mother-in-child guide extension strategies) [51].

### 3.5. Traditional Intravascular Lithotripsy (IVL)

IVL employs a balloon-based system that produces localized sonic pressure waves inside the coronary artery to selectively crack intimal and medial calcium, boosting vessel compliance and enabling optimal stent deployment [54]. The sonic pressure waves in the shape of a sphere create a field effect to treat circumferential vascular calcium at an effective pressure of about 50 atm [55]. Shockwaves from IVL on the balloon shaft radiate outward, fracturing hard calcified structures while applying minimal radial force to soft tissues, thus lowering barotrauma risk versus high-pressure NC balloons [15]. IVL safely and effectively treats heavily calcified coronary lesions with almost 100% procedural success and minimal complications [56,57,58], confirming IVL’s favorable risk profile in complex and high-risk PCI patients [59,60]. Unlike high-pressure or atherectomy devices, IVL’s low-pressure acoustic waves create controlled calcium fractures with less vessel injury, making it ideal for circumferential plaques, calcified nodules, and under-expanded stents, though deliverability remains a major limitation [56]. Real-world registries and the DISRUPT CAD program, consisting predominantly of prospective, non-randomized studies, corroborate these findings in clinical practice, reporting high procedural success rates (>92%), low MACE (<2% at 30 days), significant luminal gain, and strong stent expansion even in high-risk patients with severe calcification [61,62], with the major determinant of MACEs the larger pre-dilatation balloon diameters (*p* = 0.032) [63]. Also, in a recent propensity-score-matched comparison study of calcified coronary lesions, IVL achieved a significantly higher procedural success rate than high-pressure NC balloon angioplasty, with greater acute luminal gain and similarly low rates of angiographic complications. Despite superior procedural efficacy, 12-month MACE rates were comparable between IVL and standard high-pressure PTCA, supporting IVL as a safe and more effective lesion-preparation strategy in heavily calcified coronary disease [64]. IVL is a safe, effective, and user-friendly calcium-modification technique that improves vessel compliance and stent expansion by inducing multiplanar fractures of both superficial and deep coronary calcium, with minimal risk of barotrauma, embolization, or thermal injury [65]. Evidence supports IVL as a valuable alternative or complement to atherectomy, particularly in complex settings such as calcified left main disease, bifurcations, calcified nodules, chronic total occlusions, and stent under-expansion [66], in different clinical scenarios [6,7,67].

## 4. Limitations of Current Balloon-Based Strategies

Non-compliant, scoring, and cutting balloons mainly affect superficial calcium and plaque pliability. They frequently fail to produce the deep or circumferential fractures needed for effective stent expansion in heavily calcified lesions. Also, balloon-based approaches lack the energy needed to consistently target deep medial or nodular calcium deposits. These calcium types drive stent under-expansion and elevate restenosis risk in complex coronary artery calcification [15]. Stent under-expansion often occurs in calcified, balloon-resistant lesions, even when adequately sized NC balloons are used at high pressures (≥18 atm) but fail to fully expand the stent [68]. Aggressive high-pressure dilation using NC or UHP balloons risks coronary dissection and perforation, particularly in tortuous or fragile vessels, requiring precise sizing and imaging guidance. Cutting and scoring balloons, though they manage focal stress better, can still lead to controlled dissections or intimal damage if not sized correctly or used without real-time imaging, potentially complicating procedures and necessitating extra stenting [69,70,71]. Balloon-based devices frequently struggle to navigate tight stenoses or tortuous vessels, especially in multi-segment or extended calcified lesions, limiting their use without prior lesion modification [72,73,74]. UHP balloons and similar calcium modification techniques heighten procedural complexity, forcing operators to weigh escalating pressures against risks like dissection. This balancing act often extends procedure times relative to more precise, targeted methods [5,21,41]. Outcomes with balloon-based devices vary widely depending on lesion characteristics, operator skill, and use of imaging guidance, resulting in inconsistent results that may require switching to atherectomy, lithotripsy, or hybrid approaches [75].

## 5. Novel HC-IVL Technology

**Figure 1:**
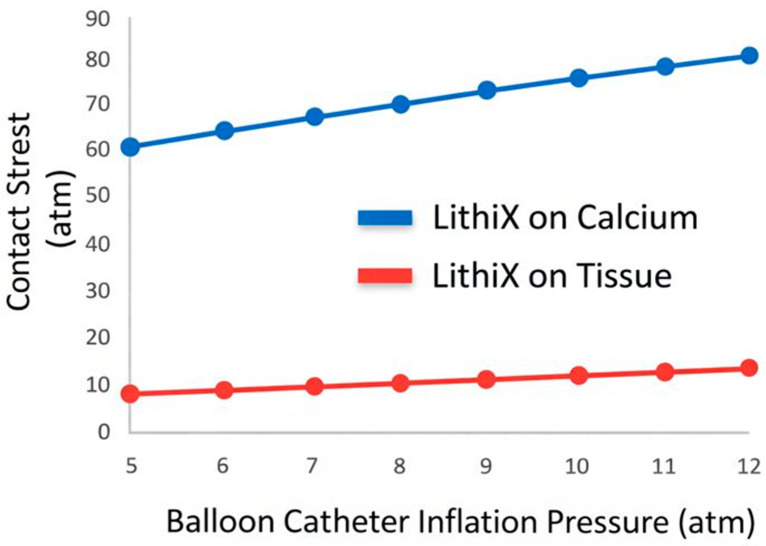
Contact stress amplification generated by HC-IVL on calcified plaque versus compliant vascular tissue. The graph illustrates the relationship between balloon catheter inflation pressure (*x*-axis, 5–12 atm) and the resulting localized contact stress (*y*-axis, expressed in atmospheres) at the interface between the device hemispheres and two different substrates: calcified plaque (blue curve) and compliant vascular tissue (red curve). As inflation pressure increases, contact stress rises proportionally; however, stress amplification is markedly greater when applied to rigid calcified tissue compared with compliant soft tissue. This differential stress distribution reflects the principles of Hertzian contact mechanics, whereby force concentration occurs over a smaller contact area in rigid materials, while stress is dissipated across a larger interface in softer tissue. The figure conceptually demonstrates the “hard-on-calcium, soft-on-tissue” effect of HC-IVL, supporting its potential for selective calcium modification at relatively low balloon inflation pressures. The values shown are schematic representations derived from contact mechanics modeling and are intended to illustrate the theoretical stress differential rather than direct in vivo measurements.

HC-IVL represents a novel mechanistic class within balloon-based calcium modification technologies. Unlike traditional approaches that rely on uniform circumferential pressure, cutting elements, or acoustic energy transmission, HC-IVL is based on the principle of Hertzian contact stress, enabling localized mechanical force expansion at the calcium–balloon interface. Hertzian contact mechanics describe the stress distribution generated when two bodies with different elastic properties come into contact over a limited surface area [76]. When a rigid object with a high modulus of elasticity contacts a harder substrate such as vascular calcium, force is concentrated over a small contact zone, resulting in marked local stress amplification within the calcified tissue. Conversely, when the same force is applied to softer, more compliant tissue, the contact area increases and stress is dissipated, thereby reducing the risk of injury. In the coronary circulation, this phenomenon enables tissue-selective energy transfer, preferentially targeting calcified plaque while minimizing stress on compliant vascular tissue (Figure 1).

**Figure 2:**
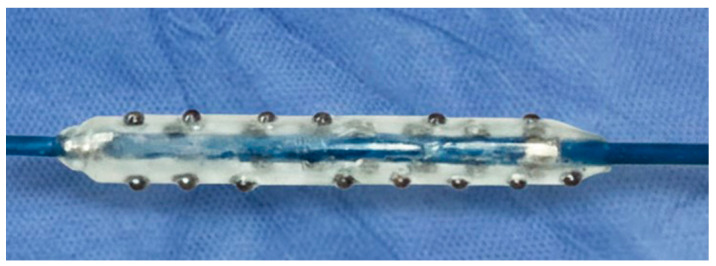
Representative image of the HC-IVL device illustrating the contact-based design with discrete metallic hemispheres mounted on the balloon surface. Unlike conventional fluid-mediated IVL systems, energy delivery occurs through direct plaque–device contact, generating localized stress concentrations at predefined contact points.

The HC-IVL system operationalizes this principle through the incorporation of multiple small metal hemispheres mounted on the surface of a semi-compliant balloon (Figure 2). During balloon inflation, each hemisphere functions as a discrete focal contact point, concentrating force directly onto the calcified plaque. A key characteristic of HC-IVL is its effectiveness at relatively low balloon inflation pressures, typically within the range used for standard balloon angioplasty (e.g., 5–12 atm). Because calcium modification is achieved through stress concentration rather than circumferential stretch, effective fracture formation does not require extreme pressures. This “hard on calcium, soft on tissue” interaction reduces uncontrolled vessel expansion and may mitigate the risk of dissection or perforation, particularly in lesions with eccentric or nodular calcium. Deliverability and procedural integration are additional defining features of the technology. The HC-IVL balloon has a low crossing profile and does not require external generators, consoles, or capital equipment (Table 1). As a result, it can be deployed within a conventional percutaneous coronary intervention workflow, with a short learning curve and minimal procedural complexity [77].

Clinical validation of this mechanistic concept has been demonstrated in the PINNACLE I study, a prospective, multicenter, first-in-human single-arm trial including 60 patients across seven sites in Belgium and the Netherlands. Clinical safety reached 98.3% and angiographic effectiveness 100% success. Less than 50% and 30% residual diameter stenosis was achieved in 100% of the lesions. Safety was confirmed with only one peri-procedural non-Q-wave myocardial infarction resulting in a 1.7% MACE event rate through 30 days. No procedural angiographic complications were recorded, including no severe dissections or perforation [78]. OCT of 32 patients were analyzed in the OCT sub-study confirmed that HC-IVL produced deep and wide calcium fractures in most treated lesions, including those with extensive calcium arc and calcified nodules (88.9% had ≥2 calcium fractures). Importantly, this fracture pattern translated into consistent luminal gain (4.0 ± 1.7 mm^2^ and 5.1 ± 1.8 mm^2^ in eccentric and concentric calcified lesions respectively) and high rates of optimal stent expansion (113.5 ± 24.2% in eccentric calcified lesions, 112.8 ± 25.4% in concentric calcified lesions and 109.7 ± 12.6% in lesions with calcium nodules), supporting the mechanistic feasibility of focal stress amplification for calcium modification. However, these findings derive from a first-in-human, single-arm experience and require confirmation in larger, randomized studies powered for clinical endpoints [79,80].

## 6. Comparative Analysis: HC-IVL vs. Traditional IVL

Although HC-IVL and traditional intravascular lithotripsy share the common objective of modifying coronary calcium to facilitate optimal stent expansion, they differ fundamentally in their physical principles, modes of energy delivery, and patterns of plaque disruption. Traditional IVL relies on the generation of acoustic pressure waves within a fluid-filled balloon. These shockwaves propagate circumferentially through the vessel wall and preferentially interact with calcified tissue, inducing microfractures through repeated pulse delivery. This mechanism is non-contact-based and depends on wave transmission across tissue interfaces, with energy distributed broadly along the vessel circumference.

In contrast, HC-IVL is a contact-based mechanical technology. Energy is delivered directly to the calcified plaque through discrete metal hemispheres, and fracture formation results from localized stress concentration rather than wave propagation. This distinction has important implications for the predictability and efficiency of calcium modification. Whereas traditional IVL produces circumferential microfractures that may vary in depth depending on calcium thickness and acoustic coupling, HC-IVL has been observed in early imaging studies to generate focal, and in some cases deeper, fractures at contact sites; however, direct comparative, imaging-guided randomized data are not yet available.

Intravascular imaging highlights qualitative differences in fracture morphology between the two approaches. Traditional IVL typically produces multiple superficial to mid-depth fractures distributed along arcs of calcium. HC-IVL, by contrast, has been shown to generate fewer but deeper and wider fractures at each contact site, with a high prevalence of multiple fractures per lesion. Notably, disruption of calcified nodules, a lesion subtype historically resistant to balloon-based technologies, has been consistently observed following HC-IVL treatment. Pressure requirements and vessel interaction further differentiate the two technologies. Traditional IVL balloons are inflated to nominal pressures to enable shockwave transmission, but adjunctive high-pressure balloon dilation is frequently required to achieve adequate luminal gain. HC-IVL achieves effective calcium fragmentation with an equivalent contact stress ranging from 60 to 80 atm at lower balloon catheter inflation pressures (5–12 atm) (Figure 1), which may theoretically reduce circumferential vessel stretch. Whether this translates into lower rates of vessel injury or improved clinical outcomes remains to be determined.

From a procedural standpoint, traditional IVL requires dedicated capital equipment and specific pulse delivery protocols, which may increase procedural complexity and resource utilization. HC-IVL, by contrast, integrates seamlessly into standard balloon-based workflows without additional equipment, offering a simplified procedural paradigm. Collectively, these differences suggest that HC-IVL should be viewed as a complementary technology rather than a replacement for traditional IVL or atherectomy. Its ability to deliver focal, deep calcium fractures at low pressure positions HC-IVL as a potentially earlier or alternative option in selected calcified lesion subsets (Table 2).

## 7. Proposed Role of HC-IVL in the Balloon-Based Calcium Modification Algorithm

**Figure 3:**
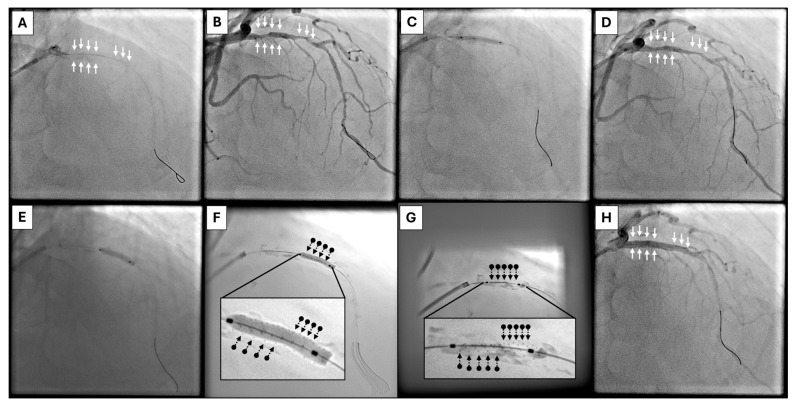
HC-IVL clinical case 1. (**A**): Severe eccentric calcification in proximal and mid LAD before contrast injection (white arrows). (**B**): Same image as in A after contrast injection, revealing severe lesions in proximal and mid LAD (white arrows). (**C**): Pre-dilatation with a NC 3.0 × 20 mm balloon sized 1:1 to the reference vessel diameter. (**D**): Image after pre-dilatation showing no significant plaque/calcium modification (white arrows). (**E**): Application of HC-IVL 3.0 × 14 mm balloon at all lesions (normal cine). (**F**): HC-IVL balloon fully inflated, as visualized in stent enhancement mode (ClearStent). The multiple small metal hemispheres mounted on the surface of the balloon can be characteristically seen as small, black dots throughout its surface (black arrow-dots). The inlet shows a magnification of the inflated HC-IVL balloon. (**G**): HC-IVL balloon during deflation, as visualized in stent enhancement mode (ClearStent). The multiple small metal hemispheres mounted on the surface of the balloon can be characteristically seen as small, black dots throughout its surface (black arrow-dots). The inlet shows a magnification of the inflated HC-IVL balloon. (**H**): Final result after stent placement (white arrows). HC-IVL: Hertz Contact Intravascular Lithotripsy, LAD: left anterior descending artery, NC: non-compliant.

**Figure 4:**
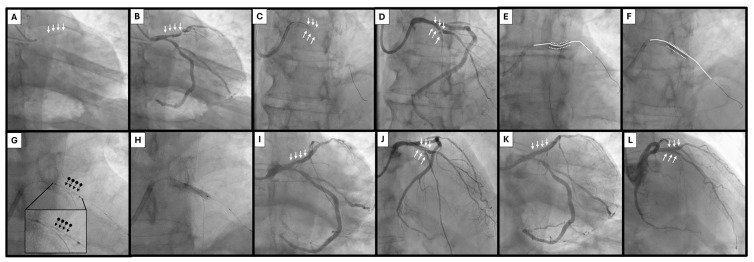
HC-IVL clinical case 2. (**A**): Severe eccentric calcification in proximal LAD before contrast injection (white arrows). (**B**): Same image as in A after contrast injection, revealing a severe lesion in proximal LAD (white arrows). (**C**): Severe eccentric calcification in mid LAD-D1 bifurcation before contrast injection (white arrows). (**D**): Same image as in C after contrast injection, revealing a severe lesion in mid LAD-D1 bifurcation (white arrows). (**E**): Predilatation of proximal LAD with a NC 3.0 × 15 mm balloon sized 1:1 to the reference vessel diameter. Notice the very eccentric balloon inflation (continuous white line: guidewire, dotted lines: balloon borders). (**F**): Predilatation of mid LAD-D1 bifurcation with a NC 2.5 × 15 mm balloon. Notice the very eccentric balloon inflation (continuous white line: guidewire, dotted lines: balloon borders). (**G**): HC-IVL 3.0 × 14 mm balloon in place at the mid LAD-D1 bifurcation lesion (normal cine). The multiple small metal. hemispheres mounted on the surface of the balloon can be characteristically seen as small, black dots around the guidewire (black arrow-dots). The inlet shows a magnification of the HC-IVL balloon. (**H**): HC-IVL 3.0 × 14 mm balloon inflated at the mid LAD-D1 bifurcation lesion (normal cine). The same balloon was applied in the proximal LAD lesion as well. (**I**): Proximal LAD image just after HC-IVL application, showing very good plaque/calcium modification (white arrows). (**J**): Mid LAD-D1 bifurcation image just after HC-IVL application, showing very good plaque/calcium modification (white arrows). (**K**): Proximal LAD final result after stent placement (white arrows). (**L**): Mid LAD-D1 bifurcation final result (white arrows) after provisional stent placement (proximal LAD to D1) and DCB application (proximal LAD to mid LAD). HC-IVL: Hertz Contact Intravascular Lithotripsy, LAD: left anterior descending artery, D1: first diagonal branch, NC: non-compliant, DCB: drug-coated balloon.

**Figure 5:**
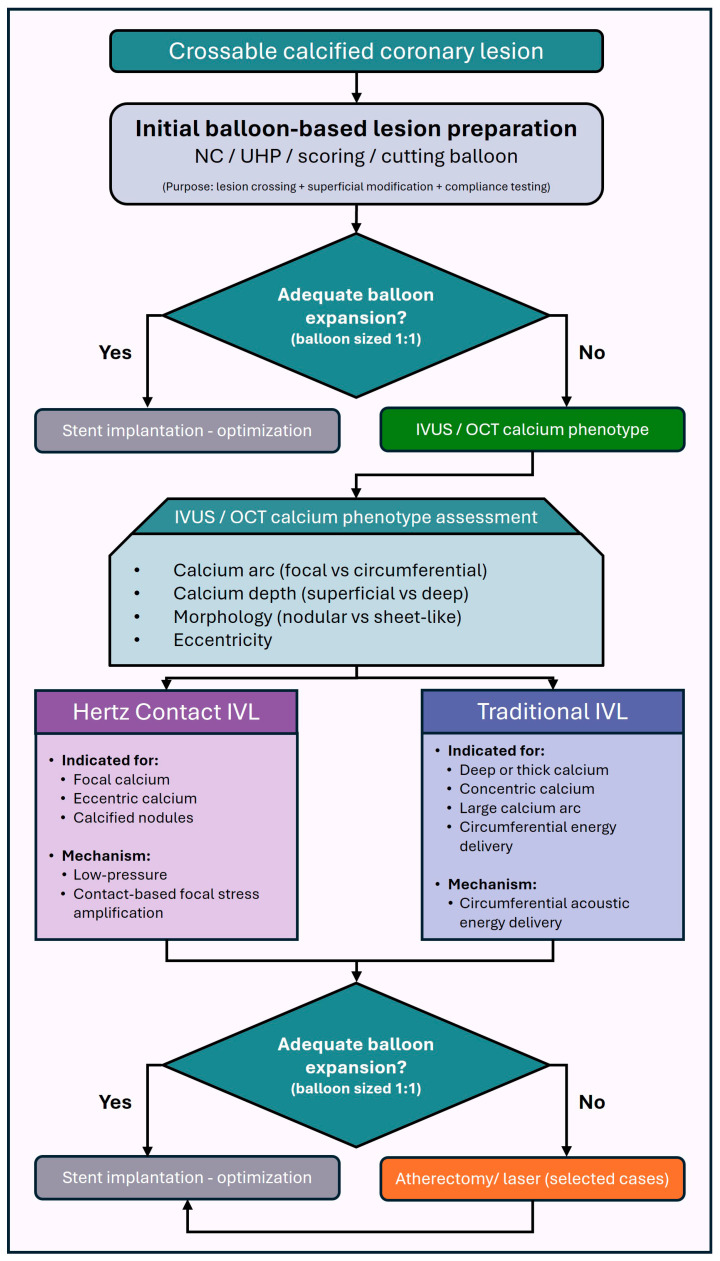
Mechanism-guided balloon-based algorithm incorporating HC-IVL. Crossable calcified coronary lesions are initially treated with non-compliant, scoring, or cutting balloons. Pre-dilatation balloons were sized 1:1 to reference vessel diameter to avoid false appearance of adequate expansion. In cases of suboptimal expansion, intravascular imaging guides further calcium modification based on calcium morphology. HC-IVL is positioned as a low-pressure, contact-based strategy for focal, eccentric, or nodular calcium, where circumferential force transmission may be inefficient. Conventional intravascular lithotripsy is reserved for concentric or deep calcium requiring circumferential energy delivery. Persistent inadequate expansion may prompt selective use of atherectomy or laser-based techniques prior to stent implantation. This algorithm represents expert-opinion-based guidance and has not yet been prospectively validated.

Contemporary treatment algorithms for calcified coronary lesions focuses on a stepwise approach integrating angiographic assessment, intravascular imaging, and escalating plaque modification strategies [81]. Balloon-based technologies remain the first-line tools for lesion preparation, with atherectomy and lithotripsy reserved for lesions resistant to conventional dilation or associated with high calcium burden. Within this framework, HC-IVL may occupy a strategic position between standard balloon-based modification and more complex plaque debulking techniques. In lesions that are crossable but demonstrate suboptimal expansion following NC, scoring, or cutting balloon inflation, HC-IVL offers an opportunity for effective calcium modification without the need for high pressures or atherectomy (Figure 3). Its low-pressure, contact-based mechanism may be particularly advantageous in eccentric or nodular calcification, where circumferential force distribution is inefficient and vessel injury risk is increased. When intravascular imaging identifies extensive calcium arc, increased calcium thickness, or nodular morphology predictive of stent under-expansion, early use of HC-IVL may improve lesion compliance in selected morphologies; however, this positioning remains conceptual and should be validated in prospective, imaging-guided comparative trials. (Figure 4 and Table 3). In this context, HC-IVL may be used either as a primary modification tool following lesion crossing or as an adjunct after inadequate balloon expansion, prior to escalation to atherectomy or laser-based techniques (Figure 5).

## 8. Future Directions

Despite substantial advances in balloon-based calcium modification, the optimal management of complex coronary calcification remains incompletely defined. The emergence of HC-IVL highlights the need for a new generation of evidence, aimed not only at procedural success but also at mechanistic validation and long-term clinical benefit. At present, HC-IVL evidence remains limited to early feasibility and mechanistic imaging data. The transition from mechanistic promise to outcome-based validation represents the critical next step in defining its clinical role. Future research should focus on defining the precise lesion subsets that derive the greatest advantage from contact-based lithotripsy, particularly in comparison with established technologies such as traditional IVL, UHP balloons, and atherectomy.

Robust clinical validation will require adequately powered, randomized controlled trials comparing HC-IVL against contemporary standards of care. Trial design should incorporate intravascular imaging-guided endpoints, including calcium fracture depth, fracture multiplicity, minimum stent area, and symmetry of stent expansion, as these mechanistic markers have been consistently linked to long-term outcomes. Importantly, patient-level clinical endpoints such as target lesion failure, restenosis, and stent thrombosis should be assessed beyond short-term follow-up, as improvements in acute luminal gain do not always translate into durable benefit. Stratification by calcium morphology (e.g., nodular versus circumferential and eccentric versus concentric patterns) will be essential to clarify whether the focal stress amplification of HC-IVL confers incremental value over circumferential shockwave-based technologies.

Importantly, most contemporary evidence supporting balloon-based calcium modification technologies, including intravascular lithotripsy and emerging contact-based approaches, is derived from non-randomized studies with lesion selection primarily based on angiographic calcification. As intravascular imaging has demonstrated, angiographically defined calcification represents a heterogeneous spectrum of calcium morphologies with distinct mechanical behaviors. This heterogeneity complicates direct comparison between devices and limits interpretation of effectiveness across trials that are not imaging guided. Future studies should therefore incorporate standardized intravascular imaging criteria to enable meaningful device comparison and morphology-specific outcome assessment.

Another important area for investigation is the potential synergy between HC-IVL and other balloon-based or debulking technologies. Sequential strategies combining HC-IVL with UHP balloons may allow controlled calcium fracture followed by uniform vessel expansion at lower pressures, potentially reducing vessel injury. Similarly, pairing scoring or cutting balloons with HC-IVL could enhance plaque modification by first creating controlled surface stress risers, followed by deep focal fracture. These hybrid approaches may be particularly attractive in long, thick, or mixed-morphology calcified lesions, where single-modality strategies frequently fail.

Advances in intravascular imaging and computational modeling are also likely to shape future development. Integration of OCT-based calcium scoring systems and real-time assessment of fracture patterns could support more personalized device selection and procedural sequencing [82]. Furthermore, finite element modeling of Hertzian contact mechanics in vivo may help refine device design, optimize hemisphere geometry, and predict fracture behavior across different calcium phenotypes. As experience grows, these insights may enable refinement of procedural algorithms that move beyond escalation by pressure alone toward mechanism-driven plaque modification.

## 9. Conclusions

Severe coronary artery calcification continues to represent a major limitation to optimal stent deployment and long-term PCI outcomes. While contemporary balloon-based technologies have expanded the interventional toolkit, important gaps remain, particularly in the treatment of thick, circumferential, eccentric, and nodular calcium. HC-IVL introduces a fundamentally different paradigm based on focal stress amplification through direct mechanical contact. By leveraging Hertzian contact mechanics, this technology enables deep and localized calcium fracture at relatively low inflation pressures, with a favorable interaction profile between calcified plaque and surrounding vessel tissue. Rather than replacing existing tools, HC-IVL may complement current calcium modification strategies. While early mechanistic and feasibility data are promising, their clinical role should be regarded as hypothesis-generating pending validation in randomized, imaging-guided trials. As imaging-guided PCI continues to evolve toward greater precision, contact-based lithotripsy offers a promising solution aligned with the principles of tailored lesion preparation. Ongoing and future studies will determine whether this novel approach can translate its mechanistic advantages into durable clinical benefit, potentially redefining the role of balloon-based technologies in the management of complex coronary calcification.

## Figures and Tables

**Table 1:** Comparison of crossing profiles and key deliverability characteristics of contemporary balloon-based calcium modification devices. The table summarizes nominal crossing profiles and design features of non-compliant (NC), scoring (OPN, Wolverine, Wedge), cutting (NaviScore), conventional intravascular lithotripsy (IVL), and HC-IVL balloons. Differences in crossing profile reflect variations in balloon architecture, surface elements, and energy-delivery mechanisms, which may influence device deliverability in severely calcified or tortuous coronary lesions. Values are based on manufacturer specifications where available.

Device	Balloon Type	Nominal Crossing Profile *	Deliverability in Severe Calcification
NC balloon	Non-compliant	Low	High
OPN NC	Ultra-high-pressure	Moderate–high	Moderate
Wolverine™	Cutting balloon	High	Limited
Naviscore™	Scoring balloon	Moderate–high	Moderate
Wedge™ NC	Scoring balloon	Moderate	Moderate
Traditional IVL	Shockwave balloon	High	Limited
HC-IVL	Contact-based lithotripsy	Low–moderate	High

* Crossing profile values are derived from manufacturer-reported specifications and may vary according to balloon diameter and device configuration.

**Table 2:** Comparison of Traditional IVL and HC-IVL. The table contrasts acoustic wave–based, non-contact IVL with contact-based HC-IVL in terms of energy delivery, plaque interaction, fracture patterns, deliverability, and procedural workflow. The comparison highlights the complementary roles of circumferential versus focal calcium modification in contemporary lesion preparation.

Feature	Traditional IVL	HC-IVL (LithiX)
**Mechanism**	Acoustic shockwave-based energy	Contact-based mechanical stress
**Plaque interaction**	Non-contact, circumferential	Direct, focal contact
**Energy distribution**	Broad circumferential dispersion	Localized stress at contact points
**Calcium fracture pattern**	Multiple, shallow circumferential fractures	Fewer, deeper focal fractures
**Effect on nodular calcium**	Limited	Consistent disruption
**Inflation pressure**	Nominal IVL; often adjunctive high-pressure dilation	Effective at lower pressures
**Workflow**	Requires dedicated generator	No need for special generator
**Deliverability**	May be challenging in tortuous or severely calcified segments	Comparable to standard semi-compliant balloons
**Clinical role**	Established for deep calcium	Complementary for focal or nodular lesions

**Table 3:** Imaging-defined calcium phenotypes and optimal balloon-based modification strategy. The table integrates IVUS- and OCT-derived calcium morphologies with their dominant mechanical constraints and the corresponding balloon-based preparation approach. Recommendations reflect mechanistic compatibility between calcium phenotype and mode of energy or force delivery.

Calcium Phenotype Based on IVUS/OCT	Key Mechanical Limitation	Preferred Balloon-Based Strategy	Rationale
Superficial, thin calcium	Limited plaque compliance	NC/scoring/cutting balloon	Direct radial force or surface incisions sufficient
Deep, concentric calcium	Global vessel constraint	Traditional IVL	Circumferential shockwaves fracture deep calcium
Focal, eccentric calcium	Localized resistance, uneven stress	HC-IVL	Focal stress amplification more efficient than circumferential force
Calcified nodules	Protruding rigid mass	HC-IVL (±scoring balloon)	Direct contact stress disrupts nodular calcium
Long, thick calcium (>5 mm, >0.5 mm)	Resistance to balloon expansion	IVL ± HC-IVL (selected cases)	Deep fracture required; focal stress may complement
Balloon un-dilatable lesion	Extreme rigidity	Atherectomy/laser	Plaque debulking required

## Data Availability

No new data were created or analyzed in this study.
